# An Overview of Meta-Analyses of Danhong Injection for Unstable Angina

**DOI:** 10.1155/2015/358028

**Published:** 2015-10-11

**Authors:** Xiaoxia Zhang, Hui Wang, Yanxu Chang, Yuefei Wang, Xiang Lei, Shufei Fu, Junhua Zhang

**Affiliations:** ^1^College of Management and Economics, Tianjin University, Tianjin 300072, China; ^2^Tianjin State Key Laboratory of Modern Chinese Medicine, Tianjin University of Traditional Chinese Medicine, Tianjin 300193, China; ^3^Institute of Traditional Chinese Medicine, Evidence-Based Medicine Center, Tianjin University of Traditional Chinese Medicine, Tianjin 300193, China

## Abstract

*Objective*. To systematically collect evidence and evaluate the effects of Danhong injection (DHI) for unstable angina (UA). *Methods*. A comprehensive search was conducted in seven electronic databases up to January 2015. The methodological and reporting quality of included studies was assessed by using AMSTAR and PRISMA. *Result*. Five articles were included. The conclusions suggest that DHI plus conventional medicine treatment was effective for UA pectoris treatment, could alleviate symptoms of angina and ameliorate electrocardiograms. Flaws of the original studies and systematic reviews weaken the strength of evidence. Limitations of the methodology quality include performing an incomprehensive literature search, lacking detailed characteristics, ignoring clinical heterogeneity, and not assessing publication bias and other forms of bias. The flaws of reporting systematic reviews included the following: not providing a structured summary, no standardized search strategy. For the pooled findings, researchers took statistical heterogeneity into consideration, but clinical and methodology heterogeneity were ignored. *Conclusion*. DHI plus conventional medicine treatment generally appears to be effective for UA treatment. However, the evidence is not hard enough due to methodological flaws in original clinical trials and systematic reviews. Furthermore, rigorous designed randomized controlled trials are also needed. The methodology and reporting quality of systematic reviews should be improved.

## 1. Introduction

**Figure 1:**
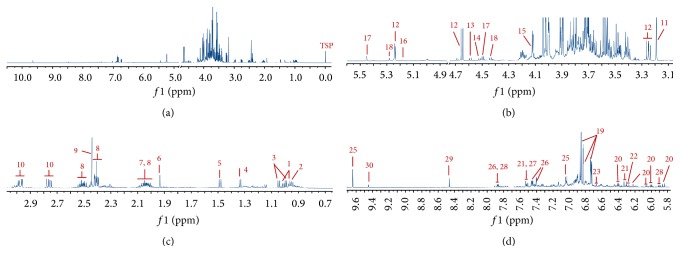
Representative ^1^H NMR spectra of DHI. Peaks: 1, isoleucine; 2, leucine 3, valine; 4, threonine; 5, alanine; 6, acetate; 7, proline; 8, pyroglutamate; 9, succinate; 10, asparagine; 11, malonate; 12, glucose; 13, galactose; 14, arabinose; 15, fructose 16, rhamnose 17, rutinose 18, rutinulose; 19, salvianic acid; 20, salvianolic acid B; 21, rosmarinic acid; 22, lithospermic acid 23, salvianolic acid A; 24, procatechuic acid; 25, procatechuic aldehyde; 26, 4-hydroxybenzoic acid; 27, 4-hydroxycinnamic acid; 28, uridine; 29, formate; 30, 5-(hydroxymethyl)-2-furaldehyde.

A report from World Health Organization indicates that ischemic heart disease is a leading cause of death in the world [1, 2]. In 2010, 2150 deaths occurred every day in the United States due to cardiovascular disease (CVD). The direct and indirect cost of CVD was USD 3154 billion in 2010 in the United States [3]. Antiplatelet drugs [4], anticoagulant [5], nitrates [6, 7], calcium channel blockers [8], and beta blockers [9] are commonly used treatments for high-risk patients with CVD. However, many patients are still not satisfied with these routine treatments. Traditional Chinese medicinal drugs have been used for cardiovascular diseases for a long time. From the perspective of traditional Chinese medicine (TCM), the pathogenesis of UA is mainly blood stagnation [10]. Danhong injection (DHI), which is typically used to resolve blood stasis [11], has been widely used in clinical practice for the treatment of UA in China. DHI is made of the extraction from Danshen (the root and rhizome of* Salvia miltiorrhiza* Bge.) and Honghua (the flower of* Carthamus tinctorius* L.). The ingredients of DHI are mainly* Salvia* phenolic acids, flavonoids safflower, benzene diene glycosides, and nucleosides. Jiang et al. [12] identified 30 compounds in DHI, including mono- and oligo-saccharide, amino acids, and low-molecular-weight organic acids (Figure 1). The quantitative nuclear magnetic resonance (qNMR) technique is utilized to quantitatively measure amino acids, mono- and oligo-saccharide, and small molecular organic acids in DHI. This enhancement in technology enables the detection of ingredients previously undetectable using the HPLC-DAD method [13, 14]. Experimental studies have shown that DHI can increase coronary blood flow [15], improve cardiac microcirculation [16], scavenge for free radicals [17], prevent platelet aggregation [13], and accommodate blood lipids [18]. Meanwhile, some studies indicated that conventional intervention plus DHI can enhance the therapeutic effect and lessen side effects of chemical drugs [19, 20]. Many clinical trials of DHI for UA have been conducted and are mainly published in Chinese journals. Furthermore, there were also some published systematic reviews/meta-analyses about DHI for UA [19, 20]. However, the quality of methodology and the conclusions of the systematic reviews/meta-analyses have not been critically assessed. This paper aimed to evaluate the quality of published systematic reviews and summarize the clinical evidence of DHI for UA.

## 2. Method

### 2.1. Inclusion Criteria

Systematic reviews of DHI for UA were included irrespective of whether meta-analysis was used. Patients should be diagnosed as UA. There were no limitations to the publishing date, language, and outcome measures.

### 2.2. Literature Searching Strategy

Seven electronic literature databases were searched to recruit candidate studies up to January 2015. Three of the databases were English databases (PubMed, Web of Science, and the Cochrane Library), and the others were Chinese-literature databases (China National Knowledge Infrastructure, Wanfang Data, sinomed, and VIP Database for Chinese technical journals). The words used for English databases were “Danhong” OR “Dan hong” OR “Danhong Injection” AND “Systematic review” OR “meta-analysis” OR “systematic reviews” OR “meta analyse.”

### 2.3. Quality Assessment

#### 2.3.1. Assessment of the Methodological Quality

AMSTAR (a measurement tool to assess the methodological quality of systematic reviews) which is a reliable and valid measurement tool for the “assessment of multiple systematic reviews” was used in this study [24, 25]. AMSTAR consists of 11 items. For each item, there are four answer options: “cannot answer,” “yes,” “no,” and “not applicable.”

#### 2.3.2. Assessment of the Reporting Quality

PRISMA (preferred reporting items for systematic reviews and meta-analyses) was used to assess the reporting quality of included systematic reviews [26, 27]. Each included review was assessed by two independent reviewers (Xiaoxia Zhang and Hui Wang). Any disagreements were resolved by discussion with other authors. The PRISMA statement consists of 27 items and aims to improve the reporting quality of meta-analyses and systematic reviews. For each item, there are three answers: “adequate,” “inadequate,” and “ inconformity.”

## 3. Results

**Figure 2:**
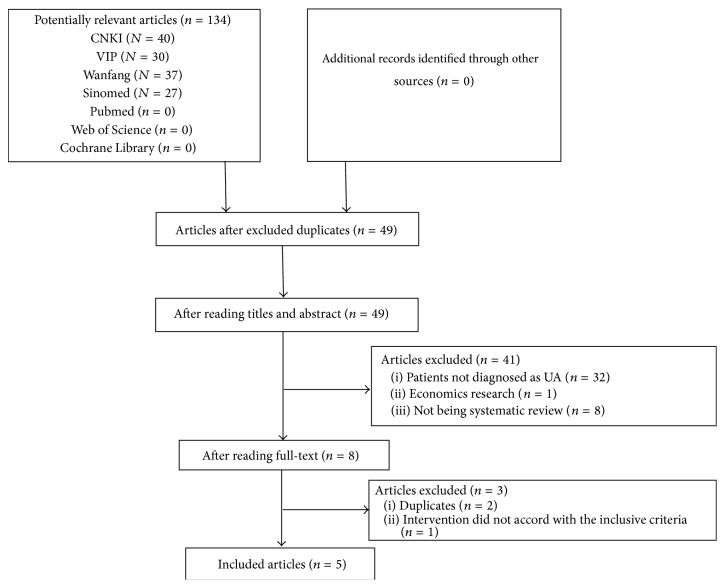
PRISMA 2009 flow diagram.

Initially, 134 articles were identified for further investigation according to the search strategy. After duplicates were removed, 49 records remained. After further screening, 41 studies were excluded according to the inclusion criteria. Three more studies were excluded after reading the full content. Finally, 5 studies were included for analyses (Figure 2).

### 3.1. Characteristics of Selected Studies

Five systematic reviews were published in Chinese from 2010 to 2012. There were 76 original studies with 7906 participants. As shown in Table 1, all included original studies are randomized clinical trials (RCT). The Jadad scores were used in 4 systematic reviews (the other one did not mention tool for quality evaluation), and most of the primary studies were of poor quality. A mean of 15 studies was included in each systematic review. The treatment courses of original studies ranged from 7 to 28 days. The main outcomes were alleviation of angina symptom and amelioration of electrocardiograms (ECG). The conclusions of the five studies were consistent, which suggest that DHI plus conventional medicine treatment was effective for UA pectoris treatment.

Wang and Hu [19] evaluated the effectiveness of DHI treatments for UA by a meta-analysis including 13 randomized controlled trials (RCTs) with 1183 participants: 623 patients in DHI treatment group and 560 patients in comparison group. The age of patients ranged from 31 to 84 years. There were 11 RCTs of DHI plus conventional treatment compared with the same conventional treatment. The remaining two RCTs were DHI compared with a 20 mL of Danshen injection and 5 mL of nitroglycerin based on conventional treatments. The meta-analysis showed that the DHI group performed significantly better than the control group in two parameters: angina symptoms (RAS) (OR = 4.98, 95% CI: 3.49~7.11) and ECG (OR = 2.48, 95% CI: 1.85~3.32). Two patients from the DHI group reported headaches, dizziness, and nausea, and 1 patient reported low blood pressure. After the drip speed of the intravenous drip was turned down, the patients recovered. There were 14 patients from the control group who reported headache. Among those 13 reports, no detailed information reported early termination or loss to follow-up. All the 13 articles were marked as low quality using the Jadad scale. Additionally, there was publication bias in the included articles.

Xu et al. [20] assessed the efficacy and safety of DHI treatments for UA in 9 RCTs, with a total of 771 participants aged 60 years or older. The meta-analysis showed that DHI plus conventional medicine was better than the conventional medicine alone in two outcome measures: RAS (OR = 3.83, 95% CI: 2.52~5.82) and ECG (OR = 2.51, 95% CI: 1.79~3.53). There was no report on adverse effects. All the included articles had low-quality grades according to the Jadad scale (score = 2). Publication bias was reported in the included articles.

Xu et al. [21] showed that DHI can effectively improve ECG (OR = 2.87, 95% CI: 2.30~3.59) and RAS (OR = 3.96, 95% CI: 3.00~5.24) in patients with UA. There were no severe adverse effects during treatment duration. There was 1 patient who appeared fatigued in DHI group. There were 2 patients who reported headaches, dizziness, and nausea in control group. Included studies were of low quality; only one reached 3 on Jadad score. There was publication bias based on the funnel plot. Few trials mentioned a randomization method or allocation concealment. Only one trial mentioned a method of blinding.

Yang et al. [22] examined 12 RCTs with 1337 participants ranging from 43 to 78 years of age. The meta-analysis showed that there was significant improvement of clinical symptoms in the DHI group compared with conventional medicine, RAS (OR = 4.01, 95% CI: 2.80~5.76). The difference between the two groups in ECG improvement was statistically significant (OR = 2.60, 95% CI: 1.98~3.41). Adverse events were not mentioned. Publication bias existed. Except for 2 trials (one that described single blinding and one that mentioned double-blinding); most of the trials did not mention blinding.

Cui et al. [23] assessed the effects of DHI on UA in 23 RCTs with a total of 2675 participants ranging from 39 to 82 years of age, and the duration of disease ranged from 21 days to 15 years. The results of the meta-analysis showed that the DHI group was superior to the control group in 5 parameters: ECG (RR = 2.84, 95% CI: 2.28~3.55), RAS (RR = 4.13, 95% CI: 3.12~5.47), increasing the serum level of HDLC (WMD = 0.29, 95% CI: 0.05~0.52), decreasing low density LDLC (WMD = −0.98, 95% CI: −1.33~0.63), and HS-CRP (WMD = −1.42, 95% CI: −2.18~−0.65). Adverse events were not mentioned, and no side effects were reported. More double-blinding RCTs with large-scale and high-quality are needed.

### 3.2. Quality of Methodology

Methodological quality of the included systematic reviews is summarized in Table 2. For the included 5 systematic reviews, none of them reported “a priori” designs. Literature searches were performed with keywords; however, the search strategy was not provided in the included reviews. Language bias existed in all 5 articles, and 2 reviews only searched Chinese databases.

All the reviews provided a list of included studies, while a list of excluded studies was not provided. The scientific quality of the included studies in formulating conclusions and methods used to combine the findings of studies were not appropriately used. A fixed-effects model was applied in 5 systematic reviews. None of the authors stated whether they included grey literature. Three (60%) of the studies showed that there was duplicate study selection and data extraction. Data were independently extracted by two researchers, and disagreements were resolved by discussion. The detailed characteristics of the included articles were provided in 3 (60%) articles. Funnel plots were applied in 3 (60%) reviews. The scientific quality of the studies were assessed and documented by 4 authors.

### 3.3. Quality of Reporting

Quality of reporting was shown in Table 3. Reporting quality of included systematic reviews was generally poor. The sections of* title, abstract,* and* introduction* were inadequately reported in all the 5 reviews.

“Systematic review” or “meta-analysis” was stated in the titles of all the reports. However, whether the studies were systematic reviews, meta-analyses, or both was not identifiable from the titles.

In the* abstract* sections, structured summary was not clearly reported in all the systematic reviews.

In Section 1, rationale of doing a systematic review was not clearly reported in all the 5 reviews.

In Section 2, three items were well reported including eligibility criteria, data collection process, and summary measures. In the items of protocol and registration, search, synthesis of results, and additional analyses were inadequately reported in 5 reviews. Eligibility criteria were stated in the 5 reviews. Five studies stated their method of data extraction and the principal summary measures. Only two reviews stated a comprehensive electronic search database, while others were lacking additional search information. All five studies did not provided search formula. There was no review describing the registration information. As for the synthesis of results and additional analysis, none of authors gave adequate descriptions. There were 3 reviews that presented the process for selecting studies and risk of bias of individual studies.

In Section 3, the reporting quality of* results of individual studies* was good. As for the three items including* study selection, synthesis of results,* and* additional analysis*, inadequate reporting was detected in 5 reviews. No review reported flow diagrams and present well-synthesized results. Clinical heterogeneity was ignored in the 5 reviews. Characteristics and risk of bias across studies were described in 3 reviews. Sensitivity analyses and subgroup analyses were not provided in all of the included studies.

In Section 4, only 1 review summarized the evidence. All researchers did not completely discuss the limitations.

Only one review presented sources of funding for the systematic review.

## 4. Discussion

### 4.1. Primary Outcomes

Five systematic reviews published from 2010 to 2012 drew the same conclusion that DHI plus conventional medicine treatment for UA is effective and safe in alleviating angina symptoms and ameliorating ECG. Four systematic reviews reported side effects, and one did not mention side effects. The rate of adverse effects is low from the included systematic reviews.

### 4.2. The Quality of Methodology and Reporting

Generally speaking, the quality of the included systematic reviews is low. The limitations of the methodology quality of those studies included the following: no a priori design was provided; incomprehensive literature searches was performed; and the search strategy was not provided in most of the included reviews. Language bias existed in all reviews. There were 3 reviews that only searched Chinese databases. None of the authors stated whether they included grey literature. Selection bias should be controlled in the processes of study selection and data extraction. In each study, at least two independent data extractors participated, and disagreements should be checked. For meta-analysis, researchers only checked the statistical heterogeneity, but clinical and methodology heterogeneity were ignored. So, the results of meta-analyses might be incorrect or meaningless.

The flaws in reporting included the following: systematic reviews and meta-analyses were not clear in the title of reviews and the main content of the article did not show up in the summary. All the five systematic reviews were published from 2010 to 2012. In accordance with the requirements of systematic reviews, the rationale of the research should be demonstrated. In addition, the necessity and significance of doing a systematic reviews should be described in the introduction. However, the follow-up studies did not mention previously published systematic reviews; that is, the follow-up studies did not fully demonstrate rationale. In conclusion, systematic reviews of some TCM were done without clinical or scientific significance, which might be due to no registration mechanism for systematic reviews in TCM. No studies specified search strategies; thus, selection bias cannot be ignored. Although flow diagrams were an ideal tool to illustrate study selection, they were not used in the five reviews.

Because not all the characteristics of studies were presented (e.g., course, follow-up period, and interventions), clinical and methodology heterogeneity were hard to evaluate. In the synthesis of results, researchers considered statistical heterogeneity, but clinical and methodology heterogeneity were ignored. Clinical and methodology heterogeneity were significant among the original clinical trials. So, dogmatic data combing with different controls, follow-up periods, and interventions would affect the result of meta-analyses. Limitations were not adequately discussed and did not deeply analyze the risk of bias such as whether the outcome and course were reasonable. The requirement of the reporting of system reviews was not strictly enforced in Chinese journals, leading to poor quality of reporting; thus, training concerning relevant knowledge is needed for journal editors.

### 4.3. Limitations of Current Evidence

The flaws in methodology and reporting weaken the results of systematic reviews. Improper use of meta-analysis will exaggerate the bias and draw the wrong conclusions. In addition to publication bias and incomprehensive search, all the included reviews were published in Chinese and reached positive conclusions, which affected the quality of the systematic reviews.

There were some limitations for this study. Inaccurate assessment of each item in AMSTAR and PRISMA may exist due to subjective judgment. Inadequate reporting of systematic reviews affected the evaluation process although inconsistencies were solved by discussion.

## 5. Conclusions

This study summarized the evidence of DHI for UA and obtained a positive result. However, poor quality of systematic reviews/meta-analyses affected reliability of current evidence. In the future, rigorous clinical trials with larger samples are needed to confirm this conclusion. The methodological and reporting quality of systematic reviews and meta-analysis should be improved.

## Figures and Tables

**Table 1:** Characteristics of included trials.

Author Year	Electronic databases	Assessment tool of primary studies	Study type	Study time	Number of trails (participants)	Experiment group (participants)	Control group (participants)	Course (d)	Main outcome	Adverse events	Authors' conclusion
Wangand Hu, 2010 [19]^※^	VIP, CNKI, and Wanfang	Jadad	RCT	2000–2009	13 (1183)	CMT plus DHI (623)	CMT (560)	7–15	Effectiveness for RAS and ECG	Yes	Effectiveness of RAS and ECG Incidence of heart event

Xu et al., 2010 [20]	Cochrane Library, Medline, Embase, CBM disc, and CNKI	Jadad	RCT	1991–2010	9 (771)	CMT plus DHI (401)	CMT (370)	10–15	Effectiveness for RAS and ECG	No	Effectiveness and safety for UA

Xu et al., 2011 [21]	Cochrane Library, Medline, Embase, CBM disc, and CNKI	Jadad	RCT	1991–2009	19 (1940)	CMT plus DHI (991)	CMT (949)	10–21	Effectiveness for RAS and ECG	Yes	Effectiveness for UA

Yang et al., 2011 [22]	CNKI, Wanfang, VIP, and CBM disc	NM	RCT	2000–2010	12 (1337)	CMT plus DHI (NM)	CMT (NM)	NM	Effectiveness for ECG and RAS	NM	Effectiveness and safety for UA

Cui et al., 2012 [23]	Pubmed, Medline, CNKI, Wanfang, and Cochrane Library	Jadad	RCT	Up to 2010	23 (2675)	CMT plus DHI (1382)	CMT (1293)	10–28	Effectiveness for ECG and RAS Regulation of HDL-C, LDL-C, and HS-CRP	NM Or no	Effectiveness for ECG Regulation of HDL-C, LDL-C, and HS-CRP

DHI: Danhong injection; NM: not mentioned; CMT: conventional medicine treatment, including the combination of platelet aggregation inhibitor (aspirin), nitrates, beta-blockers, calcium channel blockers, and ACE inhibitors; RAS: resolution of angina symptoms; ECG: electrocardiogram; HDL-C: high density lipoprotein-cholesterol; LDL-C: low density lipoprotein-cholesterol; HS-CRP: high sensitive C-reactive protein; ※ indicates the intervention of 2 trials that were conventional medicine treatment plus DHI versus conventional medicine treatment plus Danshen injection; CNKI: China National Knowledge Infrastructure; CBM disc: Chinese Biology Medicine disc.

**Table 2:** Methodological quality assessment of systems review/meta-analysis [24, 25].

AMSTAR	Yes		No		Cannot answer		Not applicable	
Items	*n*	%	*n*	%	*n*	%	*n*	%
1	0	0	5	100	0	0	0	0
2	3	60	0	0	2	40	0	0
3	0	0	0	0	5	100	0	0
4	0	0	5	100	0	0	0	0
5	0	0	5	100	0	0	0	0
6	3	60	2	40	0	0	0	0
7	4	80	1	20	0	0	0	0
8	0	0	5	100	0	0	0	0
9	0	0	5	100	0	0	0	0
10	3	60	2	40	0	0	0	0
11	1	20	4	80	0	0	0	0

(1) Was an “a priori” design provided? (2) Were there duplicate study selection and data extraction? (3) Was a comprehensive literature search performed? (4) Was the status of publication (i.e., grey literature) used as an inclusion criterion? (5) Was a list of studies (included and excluded) provided? (6) Were the characteristics of the included studies provided? (7) Was the scientific quality of the included studies assessed and documented? (8) Was the scientific quality of the included studies used appropriately in formulating conclusions? (9) Were the methods used to combine the findings of studies appropriate? (10) Was the likelihood of publication bias assessed? (11) Was the conflict of interest stated?

**Table 3:** Report quality evaluation of system review/meta-analysis.

PRISMA		Adequate		Inadequate		Inconformity	
Section/topic		*n*	%	*n*	%	*n*	%
Title	Title	0	0	5	100	0	0

Abstract	Structured summary	0	0	5	100	0	0

Introduction	Rationale	0	0	5	100	0	0
	Objectives	0	0	5	100	0	0

Methods	Protocol and registration	0	0	0	0	5	100
	Eligibility criteria	5	100	0	0	0	0
	Information sources	2	40	3	60	0	0
	Search	0	0	5	100	0	0
	Study selection	3	60	2	40	0	0
	Data collection process	5	100	0	0	0	0
	Data items	1	20	4	80	0	0
	Risk of bias in individual studies	3	60	2	40	0	0
	Summary measures	5	100	0	0	0	0
	Synthesis of results	0	0	5	100	0	0
	Risk of bias across studies	4	80	1	20	0	0
	Additional analyses	0	0	5	100	0	0

Results	Study selection	0	0	5	100	0	0
	Study characteristics	3	60	2	40	0	0
	Risk of bias within studies	3	60	2	40	0	0
	Results of individual studies	5	100	0	0	0	0
	Synthesis of results	0	0	5	100	0	0
	Risk of bias across studies	4	80	1	20	0	0
	Additional analysis	0	0	5	100	0	0

Discussion	Summary of evidence	1	20	4	80	0	0
	Limitations	0	0	5	100	0	0
	Conclusions	1	20	4	80	0	0

Funding		1	20	0	0	4	80
